# Using the Perfusion Index for Block Success in Pediatric Patients: A Retrospective Study

**DOI:** 10.7759/cureus.45281

**Published:** 2023-09-15

**Authors:** Gozde Altun, Engin Ihsan Turan, Ayca Sultan Sahin

**Affiliations:** 1 Anesthesiology and Reanimation, Istanbul University Cerrahpasa Institute of Cardiology, Istanbul, TUR; 2 Anesthesiology and Reanimation, Kanuni Sultan Suleyman Education and Training Hospital, Istanbul, TUR

**Keywords:** pediatric, anesthesia, success, block, index, perfusion

## Abstract

Study objective: To investigate the efficacy of the perfusion index in assessing block success in pediatric patients undergoing elective supracondylar fracture repair surgery.

Methods: It was a retrospective study in a tertiary-care center. Twenty-eight pediatric supracondylar humerus fracture patients who underwent elective surgery for fracture repair were evaluated. Perfusion index, pulse rate, pleth variability index (PVi), and oxygen saturation were measured at different time intervals before and after the coracoid infraclavicular block procedure.

Main results: The changes in perfusion index (PI) values were found to be statistically significant (p˂0.05). The Bonferroni analysis revealed that the results obtained at three separate measurement times differed significantly (p˂0.05). On the other hand, changes in other variables were not statistically significant (p˃0.05).

Conclusions: The perfusion index can be used as an indicator of block success in elective surgeries of the upper extremities in pediatric patients.

## Introduction

Regional anesthesia is a crucial component of modern anesthesia practice, providing analgesia and anesthesia in specific body regions while avoiding the side effects of general anesthesia [1]. Infraclavicular brachial plexus block (IC-BPB) has several advantages over other anesthesia techniques, especially in children with trauma, because of the risk of gastric aspiration with general anesthesia. Also, with the use of ultrasound, a higher success rate, a more rapid onset of anesthesia, and fewer complications are other benefits [2]. 

The IC-BPB method can be performed in various ways, including the coracoid infraclavicular, retroclavicular infraclavicular, and lateral sagittal infraclavicular approaches [3,4]. The coracoid infraclavicular block necessitates the injection of the local anesthetic around the cords of the brachial plexus. The IC-BPB is a regional anesthesia technique often used in upper extremity surgical procedures, including hand, forearm, and elbow surgeries. 

Perfusion index (PI) is a non-invasive means of measuring peripheral perfusion and is widely used in anesthesia, critical care, and pediatrics [5-8]. PI monitoring uses the principle of photoplethysmography (PPG). The determinants of the PI are vascular tone and stroke volume. However, these parameters can be affected by peripheral or central hemodynamic variables. The PI is derived from the PPG signals and reflects the pulsatile-to-non-pulsatile light absorbance ratio measured by a specific pulse oximeter. The values in the 0.2%-20% range are accepted as normal [6]. PI monitoring is based on the principle that a successful regional anesthesia block results in local vascular dilatation caused by the blockage of an autonomic nerve. Low PI indicates high peripheral vasomotor tonus, whereas high PI implies low peripheral vasomotor tone. Changes in PI value can be associated with elevated skin temperatures and increased regional perfusion [7].

Regional anesthesia in children, particularly toddlers and preschoolers, can be challenging because of the low age and sedation. Evaluation of block success with conventional methods is complex in these patients due to the lack of cooperation [9]. This study aimed to investigate PI's efficacy in assessing block success in pediatric patients undergoing elective supracondylar fracture repair surgery.

## Materials and methods

This retrospective study was initiated after approval from the institutional ethics committee of the Health Science University Istanbul Kanuni Sultan Suleyman Training and Research Hospital. It was conducted in accordance with the principles reported in the Declaration of Helsinki. Family members of all study participants consented to the use of their data for study purposes. Pediatric patients aged between 3 and 16 who underwent elective supracondylar fracture repair surgery in our institution with an American Society of Anesthesiologists (ASA) score of I or II constituted the target population of this study. The study period was a six-month period between January and June 2022. 

Patients whose family members refused participation in the study, those who had an infection at the puncture site or other contraindications for regional anesthesia, and patients with an allergy to the drugs used were excluded.

Preoperatively, all patients fasted according to the standard nil per oral (NPO) guidelines, allowing ingestion of clear fluids up to two hours before the procedure. Intravenous (IV) access was secured in the ward. Before the surgery, all patients were given 0.05 mg/kg IV midazolam as premedication. Once taken to the operating room, patients were monitored routinely by electrocardiogram (ECG) and heart rate (HR), blood pressure (BP), respiration rate (RR), and pulse oxygen saturation (SPO2) monitoring. 

All patients were placed supine and monitored with Masimo® pulse oximetry on the index finger on the side of the surgery. The working principle of Masimo® pulse oximetry is based on the ratio between the pulsatile and non-pulsatile components of the light-reaching and light-sensitive blood cells [10]. PI was measured with Masimo® pulse oximetry automatically.

During the study period, it was preferred to use the coracoid infraclavicular approach, which is routinely performed in our clinic. The patients were sedated with ketamine (1 mg/kg), and the IC-BPB was performed under sterile conditions. An 8-12 mmHz linear ultrasound (Esaote Mylab Ultrasound, China) was used. After the patients were deeply sedated, a diluted (0.3mg/kg) bupivacaine (0.25%) and lidocaine (0.5%) solution was prepared. Under ultrasound guidance, the same experienced anesthesiologist used an in-plane needle (Braun Stimuplex® Ultra 360® needle, B-Braun Medical, India) to block the infraclavicular brachial plexus in each patient. 

The PI of the patients who underwent IC-BPB was measured and recorded three times by the anesthesiologist who performed the nerve block: before the nerve block, one minute, and five minutes after the block. The block was considered successful in patients with increased PI. A 20% increase in the PI was considered significant. Pulse rate, Pleth Variability Index (PVi), and SpO2 measurements were also recorded. These surgical procedures lasted mostly 15-20 minutes. The analgesic and anesthetic effects of ketamine were sufficient for short-term procedures.

Statistical analysis: All statistical analyses were performed using IBM Corp. Released 2010. IBM SPSS Statistics for Windows, Version 19.0. Armonk, NY: IBM Corp. The analysis of variance (ANOVA) method was used for calculating the mean values of the repeated measures such as HR, SP02, PVi, and PI. A Bonferroni analysis was performed to make multiple comparisons between each independent group. The Pearson correlation method was conducted to study the correlation between drug volume, age, and kilogram values with saturation, pulse, PVi, and PI values.

The total sample size was determined based on the G power 3.1.9.4 analysis results. The effect size was 0.25, the power (1-β err prob) was 0.95, and according to this calculation, the total sample size was 28.

## Results

This study included 28 pediatric supracondylar humerus fracture patients who underwent elective surgical repair at our center between January 2022 and June 2022 (Table 1). Among these, 16 (57.1%) were male, and 12 were female (42.9%). While 13 (46.4%) patients were younger than seven, 15 (53.6%) were over seven years old. The ANOVA method was used for calculating the mean values of the repeated measures such as HR, SP02, PVi, and PI. The changes in PI values were found to be statistically significant (p˂0.05). The Bonferroni analysis revealed that the results obtained at three separate measurement times differed significantly (p˂0.05). On the other hand, changes in other variables were not statistically significant (p˃0.05) (Table 2). 

**Table 1:** Demographic data ml: milliliter, N: sample size

		N	%
Drug volume (ml)	1-10 ml	19	67.8
	Above 10 ml	9	32.2
Age	Under seven	13	46.4
	Over seven	15	53.6
Gender	Male	16	57.1
	Female	12	42.9

**Table 2:** Mean oxygen saturation, heart rate, PVi, and PI values at different time intervals min: minutes, PVi: Pleth Variability Index, PI: Perfusion Index, bpm: beat per minute, SD: standard deviation value

	Mean±SD	Median (Minimum-Maximum)	F	p
Baseline saturation (%)	98.19±1.59	98 (95-100)	1.473	0.240
Saturation 1^st^-min. (%)	98.48±1.12	99 (96-100)		
Saturation 5^th^-min. (%)	98.59±1.22	99 (96-100)		
Baseline heart rate (bpm)	105.41±23.37	106 (60-161)	1.168	0.313
Heart Rate 1^st^-min. (bpm)	106.78±21.72	107 (64-154)		
Heart Rate 5^th^-min. (bpm)	102.89±19.96	100 (60-166)		
Baseline PVi	21.81±9.9	18 (9-51)	1.739	0.196
PVi 1^st^-min.	23.41±10.75	21 (12-59)		
PVi 5^th^-min.	23.52±10.86	21 (10-59)		
Baseline PI	2.62±2.17	2.2 (0.1-7.8)	39.920	0.000
PI 1^st^-min.	4.6±3.09	4 (0.4-12)		
PI 5^th^-min.	6.47±4.41	6.6 (0.2-16.3)		

The Pearson correlation method was conducted to study the correlation between drug volume, age, and kilogram values with saturation, pulse, PVi, and PI values. As a result of this method, there was a negative correlation between drug volume and 1st-min. heart rate and 5th-min. heart rate values and a positive and moderate correlation between 1st-min. PI and 5th-min. PI values. The correlation was negative between age and baseline heart rate value (1st-min heart rate) and 5th-min heart rate, but positive between 1st-min PI value and 5th-min PI value, moderately. There was a negative correlation between kilogram values and 1st-min heart rate values and a positive, moderate, and statistically significant correlation between 1st-min PI values and 5th-min PI values. (p˂0.05) (Table 3).

**Table 3:** Correlation of drug volume, age, and kilogram values with saturation, heart rate, PVi, and PI values min: minutes, PVi: Pleth Variability Index, PI: Perfusion Index, bpm: beat per minute

	Drug volume (ml)		Age		Kilogram	
	r	p	r	p	r	p
Baseline saturation (%)	0.151	0.451	0.194	0.333	0.095	0.636
Saturation 1^st^-min. (%)	-0.128	0.526	-0.113	0.574	-0.121	0.547
Saturation 5^th^-min. (%)	-0.352	0.071	-0.358	0.067	-0.356	0.069
Baseline heart rate (bpm)	-0.285	0.150	-0.447	0.019	-0.284	0.150
Heart rate 1^st^-min. (bpm)	-0.421	0.029	-0.590	0.001	-0.414	0.032
Heart rate 5^th^-min. (bpm)	-0.396	0.041	-0.429	0.026	-0.341	0.081
Baseline PVi	-0.069	0.733	-0.009	0.964	-0.103	0.610
PVi 1^st^-min.	-0.141	0.483	-0.063	0.753	-0.169	0.399
PVi 5^th^-min.	-0.199	0.318	-0.071	0.725	-0.222	0.265
Baseline PI	0.313	0.112	0.377	0.053	0.270	0.173
PI 1^st^-min.	0.487	0.010	0.511	0.006	0.409	0.034
PI 5^th^-min.	0.579	0.002	0.594	0.001	0.506	0.007

## Discussion

The perfusion index is an early marker regarding peripheric vasodilatation [11,12]. Specifically, PI values depend on blood flow in the peripheral circulation and the vascular tone, reflecting two main determinants: cardiac output and the balance between the sympathetic and parasympathetic nervous systems. Reduced vascular tone and increased blood flow are indicators of a successful brachial plexus block.

Evaluation of block success in pediatric patients is challenging due to communication difficulties and limitations in cooperation. Other than conventional methods such as pinprick and cold tests, it is evident that using tests that objectively evaluate block success in these patients will be very helpful in anesthesia management. 

Several researchers investigated the role of PI value in anesthesia management [13]. Seyhanlı et al. analyzed the efficacy of infraclavicular block using PI. In this study, the patients were aged between 18 and 65. This study concluded that the PI value was a valuable and sensitive parameter compared with conventional methods such as the pinprick test and Modified Bromage Scale [13].

In a recent study, Rajan et al. performed a caudal block under general anesthesia for postoperative analgesia in 25 children aged between 1 and 6 years [14]. They recorded the mean arterial pressure (MAP), PI, and heart rate before and after the caudal block at different intervals, including at the time of the skin incision. These authors accepted a 15% decrease in MAP and heart rate and a 100% increase in PI as indicators of an effective block. They concluded that PI was an earlier and more sensitive indicator than heart rate and MAP. 

In our study, there was no statistically significant difference between the patients' pre-clavicular block and post-clavicular block first-minute and fifth-minute SpO2 and HR values. We suggest that our approach, including the premedication performed in these pediatric patients, the assignment of an anesthesiologist experienced in ultrasound-guided block procedures, the administration of the anesthetic to the right site with the appropriate dose, and the selection of the patients with ASA I or II scores, significantly contributed to this finding. 

In another study using PI to assess block success, Cetgen et al. found that PI was an effective method for evaluating axillary block success in patients aged between 18 and 65 [15]. Similarly, Bereket et al. compared the efficacies of PI, resistance index, end-diastolic velocity, brachial artery diameter, peak systolic velocity, and time-average velocity in evaluating the success of infraclavicular block and found that PI, resistance index, and end-diastolic velocity were more accurate indicators [16]. 

Abdelnasser et al. worked on PI to assess block success in patients undergoing supraclavicular block in a prospective study including 77 patients [17]. They concluded that an increase of 1.4-fold or more indicated block success. Kus et al., who performed an infraclavicular block on their patients, reported that the block procedure led to a significant change in PI in the first 10 minutes [18].

Although several studies showed that PI was an indicator of block success, most of these studies were conducted with an adult patient population [17,18]. Our study revealed that each pediatric patient had a different baseline (i.e., pre-block) PI value, which should be determined before the block for comparison with the subsequent values measured at different time intervals after the procedure. In our analysis, the comparison of the baseline, post-block 1-minute, and 5-minute PI values revealed a statistically significant increase. However, a similar comparison regarding PVi did not show a statistically significant difference. Thus, it can be suggested that PVi was ineffective in evaluating block success. As such, HR was found to be insufficient to predict block success, according to our results. The fact that HR is affected by many factors, such as fluid status, stress, and arrhythmia, should be considered while evaluating this finding [17,18]. In addition to that, using only coracoid approaches for IC-BPB could be a limitation for this study. Other approaches may reveal different findings.

## Conclusions

In the literature, there is consensus on the suggestion that PI effectively indicates block success. However, it should be noted that the PI value was measured in relatively wide time intervals, such as 10, 15, or 20 minutes. In our study, we performed the PI measurements at shorter intervals (i.e., 1 and 5 minutes). We also found that PI was still an effective indicator of block success with this approach. We believe administering a short-acting local anesthetic leading to rapid-onset anesthesia contributed to this finding. Therefore, PI can be used as an indicator of block success in elective surgeries of the upper extremities in pediatric patients and is a time-saving method.
